# Isolation methods determine human neutrophil responses after stimulation

**DOI:** 10.3389/fimmu.2023.1301183

**Published:** 2023-11-24

**Authors:** Vanessa Krémer, Ophélie Godon, Pierre Bruhns, Friederike Jönsson, Luc de Chaisemartin

**Affiliations:** ^1^ Institut Pasteur, Université Paris-Cité, Institut national de la santé et de la recherche (INSERM) Unité mixte de recherche (UMR)1222, Antibodies in Therapy and Pathology, Paris, France; ^2^ Inflammation, Microbiome and Immunosurveillance, INSERM, Université Paris-Saclay, Orsay, France; ^3^ Centre national de la recherche scientifique (CNRS), Paris, France; ^4^ L'Assistance Publique - Hôpitaux de Paris (APHP), Bichat Hospital, Immunology Department, Paris, France

**Keywords:** neutrophil (PMN), isolation, human blood, phenotype, functional analysis

## Abstract

Studying neutrophils is challenging due to their limited lifespan, inability to proliferate, and resistance to genetic manipulation. Neutrophils can sense various cues, making them susceptible to activation by blood collection techniques, storage conditions, RBC lysis, and the isolation procedure itself. Here we assessed the impact of the five most used methods for neutrophil isolation on neutrophil yield, purity, activation status and responsiveness. We monitored surface markers, reactive oxygen species production, and DNA release as a surrogate for neutrophil extracellular trap (NET) formation. Our results show that neutrophils isolated by negative immunomagnetic selection and density gradient methods, without RBC lysis, resembled untouched neutrophils in whole blood. They were also less activated and more responsive to milder stimuli in functional assays compared to neutrophils obtained using density gradients requiring RBC lysis. Our study highlights the importance of selecting the appropriate method for studying neutrophils, and underscores the need for standardizing isolation protocols to facilitate neutrophil subset characterization and inter-study comparisons.

## Introduction

Neutrophils are the predominant leukocyte population in human blood and maintain host immunity through a broad arsenal of antimicrobial activities. Consequently, deficiencies in neutrophil counts or their dysfunction render individuals susceptible to recurrent and overwhelming infections (1, 2).

Upon infection, neutrophils are recruited to sites of inflammation in a tightly regulated process designed to prevent their inappropriate activation, which can lead to severe tissue and organ damage. To eliminate invading pathogens, neutrophils possess a variety of antimicrobial effector mechanisms, including phagocytosis, production of reactive oxygen species (ROS), release of preformed lytic enzymes, antimicrobial peptides, cytokines, and neutrophil extracellular traps (NETs) (3–5). While beneficial to the host in fighting infection, these same effector mechanisms also enable neutrophils to contribute to various inflammatory and autoimmune diseases (6, 7). Understanding the fine equilibrium between the beneficial and detrimental functions of neutrophils is critical to harnessing their therapeutic potential (8).

Under steady-state conditions, 10^11^ mature neutrophils are released daily from the bone marrow into the circulation (9, 10). These cells do not divide and are short-lived (11), making them notoriously difficult to study *ex vivo* and precluding genetic manipulation. Although a few cell lines exhibit partial neutrophil characteristics, none recapitulate their full cellular phenotype and functions (12, 13). Consequently, the current gold standard for studying human neutrophil function is their timely isolation from fresh blood, followed by *ex vivo* stimulation. This approach assumes that the isolation maintains neutrophils in the same phenotypic state as they are found inside the circulation. However, neutrophils are highly sensitive to external cues and are easily activated by blood collection methods, temperature changes (14), or the isolation process itself (15, 16). Even quiescent *ex vivo* neutrophils spontaneously activate, making storage time prior to processing a critical parameter for neutrophil isolation and investigation (16–18). These variables can distort the neutrophil phenotype, leading to conflicting results on activation processes and subpopulations, and an unclear picture of the human neutrophil behavior in physiological and pathological states.

Various methods, including magnetic separation and density gradients, allow neutrophil isolation from whole blood. While flow cytometry and microfluidic techniques have emerged (19, 20), technical hurdles limit their use. Given the importance of the activation state of neutrophils for understanding their biology, there is a critical need to thoroughly characterize the impact of the isolation process on subsequent experiments to facilitate neutrophil characterization, subset identification, and comparison between studies.

In this study, we examined the five most common methods of neutrophil isolation for their efficacy and impact on neutrophil physiology: 1) dextran sedimentation followed by density gradient centrifugation with Ficoll and hypertonic lysis of erythrocytes, 2) density gradient centrifugation with Polymorphprep followed by hypertonic lysis of erythrocytes, 3) density gradient centrifugation with Histopaque-1119, followed by a second density gradient centrifugation with Percoll, and two negative selection immunomagnetic bead-based kits from different suppliers 4) Miltenyi Biotec and 5) Stemcell Technologies (**Supplementary Figure S1**).

Analyses of neutrophil yield, purity, activation and responsiveness highlighted that immunomagnetic bead or density gradient separation methods without RBC lysis yielded neutrophils phenotypically resembling blood neutrophils. Moreover, immunomagnetically isolated neutrophils displayed reduced baseline activation and increased responsiveness to weak stimuli in ROS and lytic cell death assays, in contrast to density gradient-isolated neutrophils. Our study underscores the importance of selecting the appropriate neutrophil isolation method for accurate *ex vivo* analysis.

## Materials and methods

### Human samples

Human peripheral blood samples were collected from healthy volunteers through the ICAReB platform (Clinical Investigation & Access to Research Bioresources) from the Center for Translational Science, Institut Pasteur (21). All participants received oral and written information about the research and gave written informed consent in the frame of the healthy volunteers CoSImmGEn cohort (Clinical trials: NCT 03925272), after approval of the “CPP Ile-de-France I” ethics committee).

### Neutrophil isolation using immunomagnetic beads

The EasySep Direct Human Neutrophil Isolation Kit (Stemcell Technologies) was used according to the manufacturer’s instructions. After negative selection in a magnetic field, the collected neutrophils were washed with Hank’s balanced salt solution (HBSS) and adjusted to an appropriate volume in HBSS (Ca^2+^/Mg^2+^) + 2% fetal calf serum (FCS).

Neutrophils were separated by negative magnetic selection (MACSxpress, Miltenyi Biotec, M) according to the manufacturer’s instructions. Following negative selection, the neutrophil-enriched suspension was collected, and residual erythrocytes were removed using the MACSxpress Erythrocyte Depletion Kit (Miltenyi Biotec). The resulting neutrophil suspension was washed with HBSS and resuspended to an appropriate volume in HBSS (Ca^2+^/Mg^2+^) + 2% FCS.

### Histopaque/Percoll purification

Isolation was performed as previously published (22). Briefly, 5 ml of EDTA-anticoagulated whole blood were carefully layered on top of 5 ml 1.119 g ml^-1^ Histopaque (Sigma-Aldrich) and spun for 20 min at 800g without brake. The diffuse red phase of Histopaque-1119 above the erythrocyte pellet was collected in a new tube, washed, and loaded onto a 10 ml density gradient consisting of successive layers of 65, 70, 75, 80, and 85% Percoll (GE Healthcare) diluted 10:1. After centrifugation (20 min, 800g without brake), the interface between the 75% and the 65% layer was collected, washed, and the cell concentration was adjusted to an appropriate volume in HBSS (Ca^2+^/Mg^2+^) + 2% FCS.

### Polymorphprep purification followed by RBC lysis

Whole blood was layered in a 1:1 volume ratio on top of 5 ml of Polymorphprep (Alere Technologies AS) and spun for 45 min at 500g without brake. The neutrophil phase was collected according to the manufacturer’s instructions and washed. Residual erythrocytes were removed by incubation with BD Pharm Lyse buffer (BD Bioscience) for 2 min. Cells were washed and adjusted to an appropriate volume in HBSS (Ca^2+^/Mg^2+^) + 2% FCS.

### Dextran/Ficoll purification followed by RBC lysis

The isolation was performed as previously published (23). 5 ml of blood were carefully combined with 3.3 ml of 5% Dextran (Sigma-Aldrich) and 5 ml Phosphate Buffered Saline (PBS) without Ca^2+^ and Mg^2+^ in a 15 ml tube and left untouched for 30 min to separate leukocytes from erythrocytes by sedimentation. Following separation into two phases, the upper phase was collected and slowly placed on top of 3.3 ml Ficoll-Paque Plus (GE Healthcare) into a new tube, to separate polymorphonuclear from mononuclear cells and erythrocytes. The gradient was centrifuged for 20 min at 600g without brake. Contaminating erythrocytes were removed by resuspending the cell pellet in 5 ml of BD Pharm Lyse buffer (BD Bioscience) for 2 min. Cells were washed with HBSS and resuspended an appropriate volume in HBSS (Ca^2+^/Mg^2+^) + 2% FCS.

### Phenotypic analysis of neutrophils from whole blood

Whole blood was carefully mixed in a 1:1 volume ratio with PBS and centrifuged for 5 min at 500g. The supernatant was discarded followed by the addition of 1 volume of PBS. 100 μl of this washed whole blood was incubated with Live/Dead Fixable Blue Dead Cell Stain Kit UV (Thermo Fisher Scientific) with either True Stain Monocyte blocker (BioLegend) or FcR blocker (Human True Stain FcX, BioLegend) for 15 min in the dark. Subsequently, antibody mixes (**Supplementary Table S1**) were added for 20 min in the dark. After washing, samples were resuspended in 1.8 ml 1-step fix/lyse buffer (FACS Lysing solution, BD BioSciences) and incubated for 15 min. The cells were washed and resuspended in MACS buffer (Miltenyi Biotec) and aquired on a MACSQuant 16 Analyzer (Miltenyi Biotec), prior to analysis of data using FlowJo 10.6.2.

### Analysis of purity, quantity and phenotype of isolated neutrophils

2 x 10^4^ isolated neutrophils were incubated with diluted Live/Dead Fixable Blue Dead Cell Stain Kit UV (Thermo Fisher Scientific) in combination with 1:100 True Stain Monocyte blocker (BioLegend), and for FcRs panels with 1:100 FcR block (Human True Stain FcX, BioLegend) for 15 min in the dark. Cells were washed once with HBSS and stained with antibody mixes (**Supplementary Table S1**) for 20 min in the dark. Cells were fixed for 15 min (IC Fixation Buffer, BD BioSciences), washed and resuspended with MACS buffer before acquisition. MFI was acquired on a MACSQuant 16 Analyzer (Miltenyi Biotec), and analysis of data was done on FlowJo 10.6.2. Contaminating cells were determined as the percentages of lymphocytes (CD3^pos^, CD19^pos^, CD56^pos^), monocytes (CD14^pos^), eosinophils (CD193^pos^SSC^high^) and basophils (CD193^pos^/SSC^low^). Experiments that resulted in less than 70% pure neutrophils were considered flawed and excluded from the analysis (2 donors for method Pol, 3 donors for method D/F). Neutrophil yield was calculated as the obtained cell concentration divided by the initial whole blood neutrophil input measured by flow cytometry.

### Preparation of immobilized immune complexes

Plate-bound immune complexes (ICs) were formed by using human anti-TNP IgG1 monoclonal antibodies (mAbs, in house) with homemade TNP_32_-BSA as described previously (24). Briefly, 30 μg ml^-1^ TNP_32_-BSA in 50 mM carbonate/bicarbonate buffer (pH 9.6) was coated onto a 96-well ViewPlate (Perkin Elmer) for 1 hour at 37°C. After two washes with media (HBSS + 2% FCS), 100 μg ml^-1^ of human anti-TNP IgG_1_ diluted in the same media was incubated for 1 h at 37°C. Finally, wells were washed twice with HBSS (Ca^2+^/Mg^2+^) + 2% FCS.

### CD32A blocking

Neutrophils purified by magnetic isolation (Stemcell and Miltenyi kit) were preincubated for 30 min at 37°C in HBSS (Ca^2+^/Mg^2+^) + 2% FCS supplemented with 20 μg ml^-1^ of a human anti-CD32A mAb (clone IV.3 expressed as a human IgG1 mutated in its Fc domain (N_297_D) to abrogate Fc effector function, in house) or without as control, prior exposure to plate-bound ICs. Intra- and extracellular ROS production and cell death were measured over time as described below.

### Detection of intra- and extracellular ROS

ROS production was measured by chemiluminescence as previously described (25). Briefly, 5 x 10^4^ neutrophils in 200 μl were incubated with or without stimuli (25 nM PMA (Sigma-Aldrich), 5 μM A23187 (Sigma-Aldrich), 50 μg ml^-1^ PGN (Sigma-Aldrich), 10 nM TNFα (Miltenyi Biotec), 10 μg ml^-1^ LPS (*K. pneumoniae*, Sigma-Aldrich), or plate-bound ICs for 30 min at 37°C in the presence of 50 μM luminol (Sigma-Aldrich) in a 96-well ViewPlate (Perkin Elmer). Chemiluminescence was measured every 130s using a Spark microplate reader (Tecan), with timepoint1 being the first measurement immediately after addition of stimuli, and a delta luminescence was calculated for each sample tested in duplicates as:

Equation (1):


(1) $$\Delta \text{Luminescence}=\text{Lum}{\left(\text{stimuli condition}\right)}_{\text{max}}-\text{Lum}{\left(\text{buffer condition}\right)}_{\text{timepoint}1}-\text{ Lum}{\left(\text{buffer condition}\right)}_{\text{max}}$$

### Lytic cell death measurement

Cell death was measured as loss of cellular integrity by increase in Sytox Green fluorescence, a membrane-impermeable DNA intercalator that stains extracellular DNA and DNA from cells with compromised plasma membranes, as previously described (26). Briefly, 5 x 10^4^ neutrophils in 200 μl were incubated with or without 25 nM PMA (Sigma-Aldrich), 5 μM A23187 (Sigma-Aldrich), 10 μg ml^-1^ peptidoglycan (Sigma-Aldrich), 10 nM TNFα (Miltenyi Biotec), 10 μg ml^-1^ LPS (*K. pneumoniae*, Sigma-Aldrich), or plate-bound ICs in the presence of 5 µM of Sytox Green (Thermo Fisher Scientific) in a 96-well ViewPlate (Perkin Elmer). Fluorescence was measured every 15 min for 5 h on a microplate reader (Spark, Tecan) with timepoint1 being the first measurement immediately after addition of stimuli, and delta fluorescence was calculated using Eq. (2) for each sample tested in duplicates:

Equation (2):


(2) $$\Delta \text{Fluorescence}=\text{Fluo}{\left(\text{stimuli condition}\right)}_{\text{max}}-\text{ Fluo}{\left(\text{buffer condition}\right)}_{\text{timepoint}1}-\text{ Fluo}{\left(\text{buffer condition}\right)}_{\text{max}}$$

### Statistical analyses

The statistical analysis and data visualization were performed using RStudio and GraphPad Prism V9. Normality of the data was assessed using the Shapiro-Wilk test. The appropriate statistical test was chosen based on the analysis conducted, as specified in the figure legends and text. The *P* values were reported using standard notation: ****P< 0.0001, ***P< 0.001, **P< 0.01, and *P< 0.05. All outliers were included in the data and all data points are shown in the figures.

## Results

### Neutrophil recovery, viability, and purity

We compared the performance and impact of the five most commonly used methods for isolating human neutrophils from blood: two negative selection immunomagnetic bead-based kits from different suppliers, 1) Stemcell Technologies (termed “S” hereafter) and 2) Miltenyi Biotec (“M”); 3) density gradient centrifugation with Histopaque-1119, followed by a second density gradient centrifugation with Percoll (“H/P”); 4) density gradient centrifugation with Polymorphprep followed by RBC lysis (“Pol”), and 5) dextran sedimentation followed by density gradient centrifugation with Ficoll and RBC lysis (“D/F”) (**Supplementary Figure S1**). These purification methods differ in the number of processing steps, RBC lysis requirements, chemical compounds used, price, and total time required (**Supplementary Table S2**).

**Figure 1:**
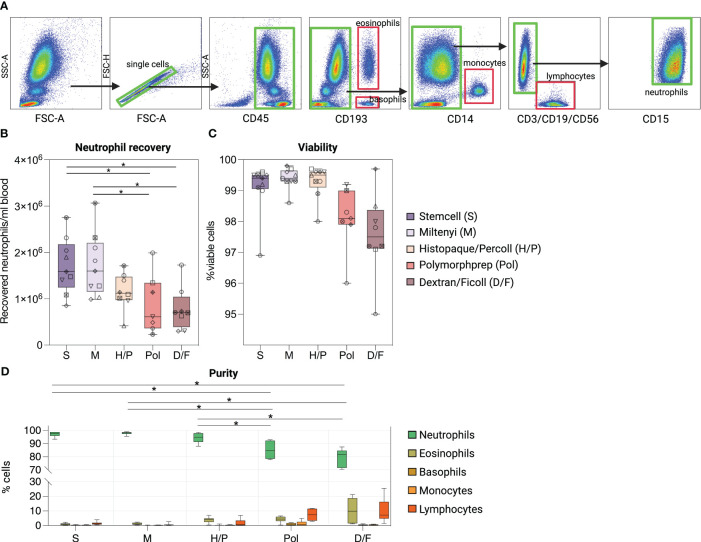
Comparison of five methods for the purification of human peripheral blood neutrophils. **(A)** Flow cytometry gating strategy to assess the viability, purity and quantity of whole blood neutrophils isolated by five different neutrophil isolation methods. Composition of isolated cell suspensions: neutrophils (SSC^high^/CD45^pos^/CD11b^pos^/CD15^pos^), eosinophils (CD193^pos/^SSC^high^), basophils (CD193^pos/^SSC^low^), monocytes (CD14^pos^) and lymphocytes [T cells (CD3^pos^), B cells (CD19^pos^) and NK cells (CD56^pos^)]. **(B)** Recovery of neutrophils per ml of blood according to the isolation method used. **(C)** Percentages of viable neutrophils after each isolation method. **(D)** Percentages of cell types identified after each isolation procedure. **(B–D)** Data are represented as boxplots; whiskers range from the minimum to maximum value and inside each box (25th to 75th percentile) the median is represented; n = 6-9. **(B, C)** Individual donors are indicated with distinct symbols. Results obtained by the different isolation methods were compared using one-way ANOVA with *post-hoc* Tukey’s multiple comparisons. *P* values are shown as *P < 0.05.

We first investigated whether the different methods affected the overall recovery of neutrophils from whole blood using flow cytometry (**Figure 1A**). Our results indicate that the immunomagnetic methods S and M consistently yielded the highest mean neutrophil recovery per ml of whole blood, whereas the density gradient methods Pol and D/F performed with 1.95-fold (Pol) and 2.02-fold (D/F) less efficiency (**Figure 1B**, **Supplementary Table S2**). All methods resulted in more than 95% viable neutrophil fractions, with a non-significant trend for the Pol and D/F methods to reduce viability compared to S, M and H/P methods (**Figure 1C**).

We then evaluated the purity of the isolated neutrophil fraction of each method by gating on SSC-A^high^/singlets/CD45^pos^/CD15^pos^ cells and assessing contamination with lymphocytes, monocytes, eosinophils, and basophils (**Figure 1D**). The immunomagnetic methods S and M as well as the density gradient method H/P showed significantly higher neutrophil purity [97.22% ± 1.77% (S), 97. 91% ± 1.06% (M), and 94.10% ± 3.61% (H/P)] than the two density gradient methods with RBC lysis Pol and D/F [78.96% ± 6.68% (Pol), 85.12% ± 6.78% (D/F)]. The major contaminating cell types in the neutrophil fractions obtained by the Pol and D/F method were lymphocytes [7.40% ± 4.16% (Pol), 9.98% ± 8.29% (D/F)] and eosinophils [4.28% ± 2.16% (Pol), 9.43% ± 8.04% (D/F)].

Overall, these results show that the immunomagnetic isolation methods are significantly superior to the density gradient methods in terms of yield and purity of the isolated fraction.

### Phenotypic analysis of isolated neutrophils

We next examined whether the different isolation methods altered the phenotype of purified neutrophils using multicolor flow cytometry. Mature neutrophils were gated as SSC-A^high^ single cells CD45^pos^CD193^neg^ expressing CD15 and excluding contaminating cell types as described previously.

**Figure 2:**
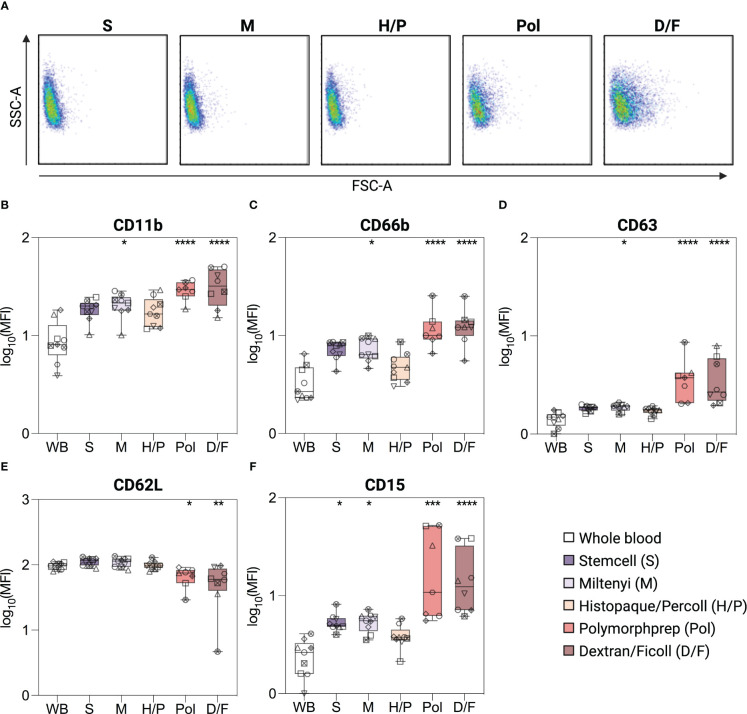
Phenotype of isolated and unprocessed whole blood neutrophils. **(A)** Size and granularity (SSC-A/FSC-A) of neutrophils according to the indicated isolation methods. **(B–F)** Surface expression of CD11b, CD66b, CD63, CD62L and CD15 on unprocessed whole blood (WB) neutrophils, or neutrophils isolated by different isolation methods. Neutrophils were gated as SSC-A^high^/singlets/CD45^pos^/CD15^pos^ cells. Data are presented as mean fluorescence intensity (MFI). Data are represented as boxplots; whiskers range from the minimum to maximum value and inside each box (25th to 75th percentile) the median is represented; n = 6-9. Individual donors are indicated with distinct symbols. Purification methods were compared with unprocessed whole blood neutrophils using one-way ANOVA with *post hoc* Tukey’s multiple comparisons or Kruskal-Wallis test followed by Dunn’s post test. *P* values are expressed as *P< 0.05, **P< 0.01, ***P< 0.001, and ****P< 0.0001.

Morphological analysis (FSC-A/SSC-A) showed that neutrophils isolated by the Pol and D/F density gradient separation methods exhibited a wider range of size and granularity compared to the three other methods or unprocessed whole blood neutrophils (**Figure 2A**). Neutrophils isolated using Pol and D/F methods also showed a shift toward a higher FSC-A and lower SSC-A, indicating an activated state (27–29).

Subsequently, a phenotypic assessment of the neutrophils was conducted to evaluate potential alterations in pertinent surface proteins, focusing on activation and degranulation markers. In the absence of a better reference, unprocessed whole blood (WB) neutrophils were used as a reference point for the “*in vivo*” status of neutrophils, using a method previously shown to have minimal impact on neutrophil phenotype [(17), see Methods]. Neutrophils isolated by density gradient methods with RBC lysis (Pol and D/F) showed a significant upregulation of the classical activation and degranulation markers CD11b, CD66b, and CD63 [2.2-, 2.6-, and 2.7-fold (Pol); 2.6-, 2.8-, and 2.8-fold (D/F)], and a downregulation of CD62L [0.5-fold (Pol, D/F)] compared to unprocessed whole blood neutrophils (**Figures 2B–E**). This phenotype is indicative of neutrophil activation (30). The Miltenyi immunomagnetic method (M) also resulted in a moderate increase in neutrophil CD11b, CD66b and CD63 (1.8-, 1.8-, and 1.3-fold), whereas CD62L expression remained similar to that of unprocessed whole blood neutrophils, indicating a milder neutrophil activation (**Figures 2B–E**). Only neutrophils isolated by the immunomagnetic method from stem cells (S) and the density gradient method Histopaque/Percoll (H/P) showed no significant increase in CD66b, CD11b and CD63 or decrease in CD62L compared to unprocessed blood neutrophils (**Figures 2B–E**).

In addition, all methods except H/P induced an increase in CD15, with a 1.8-fold increase in the S and M methods and a 6.6-/5.1-fold increase in the Pol and D/F methods (**Figure 2F**), which, although not classically used, is also indicative of neutrophil activation (31).

Taken together, our data show that isolation protocols are widely different in their alteration of neutrophil phenotype, with H/P having no effect, S and M having moderate effects, and Pol and D/F having large effects on all activation markers analyzed.

### Characterization of FcγRs on isolated neutrophils

**Figure 3:**
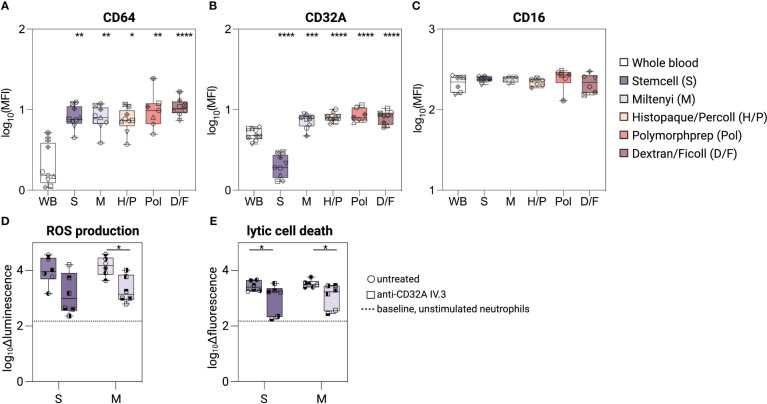
Characterization of FcγR on isolated neutrophils. **(A–C)** Surface expression level of CD64, CD32A, and CD16 on unprocessed whole blood neutrophils, or neutrophils isolated with different isolation methods. **(D, E)** Neutrophils isolated with the Stemcell or Miltenyi kit were pre-treated with anti-CD32A blocking mAb (IV.3) or not, and incubated with plate-bound human IgG1-ICs. **(D)** Delta luminescence values of intra-/extracellular ROS assay. **(E)** Delta fluorescent values of Sytox green intensity as readout for lytic cell death. Data are represented as boxplots; whiskers range from the minimum to maximum value and inside each box (25th to 75th percentile) the median is represented; n = 6-9. Individual donors are indicated with distinct symbols. Purification methods were compared to untreated neutrophils from whole blood using a one-way ANOVA with *post-hoc* Tukey multiple comparisons or Kruskal-Wallis test followed by Dunn post-test. Unblocked and blocked neutrophils were compared using Wilcoxon signed-rank test. *P* values are represented as ****P< 0.0001, ***P< 0.001, **P< 0.01, and *P< 0.05.

Three different IgG receptors (FcγR) are expressed on the surface of neutrophils, CD64 (FcγRI), CD32A (FcγRIIA) and CD16B (FcγRIIIB), and contribute to their ability to become activated and/or to phagocytose immune complexes or opsonized cells (24, 32, 33). Following isolation, CD64 surface staining was similarly increased (3.5-/4.6-fold) on neutrophils compared to unprocessed whole blood neutrophils, regardless of the isolation method used (**Figure 3A**). The same was observed for CD32A (1.3-fold) for all methods except using the Stemcell kit (S) (**Figure 3B**). Neutrophils isolated by the latter method showed a 0.3-fold reduced CD32A expression compared to unprocessed whole blood neutrophils. We confirmed these results using several other clones of CD32A mAbs (**Supplementary Figure S2**). Finally, no significant difference in CD16B expression was detected between neutrophils purified by the different methods and unprocessed whole blood neutrophils (**Figure 3C**).

To determine whether the reduction in CD32A levels caused by the Stemcell kit alters the response of neutrophils to immune complexes, we stimulated neutrophils - isolated using either the Stemcell (S) or the Miltenyi (M) kit - with plate-bound human IgG1 immune complexes. Neutrophils obtained with both methods showed similar and robust ROS release and lytic cell death induction (**Figures 3D, E**). The responses to immune complexes were mostly CD32A-dependent, as pre-incubation of neutrophils with a CD32A-blocking antibody (mAb IV.3, 34) similarly reduced ROS release and lytic cell death (**Figures 3D, E**), although failing to reach significance for the ROS measurement in neutrophils isolated with method S.

Thus, despite the low CD32A surface staining, neutrophils isolated with the Stemcell kit retain their responsiveness to IgG immune complexes.

### RBC lysis decreases ROS production capacity of isolated neutrophils

**Figure 4:**
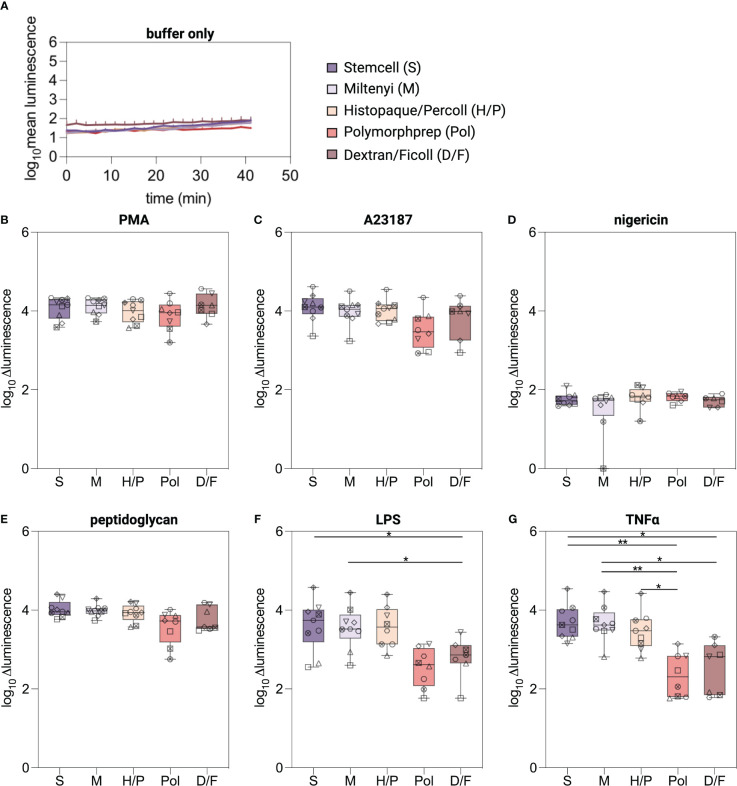
ROS production in isolated neutrophils. **(A–G)** Intra-/extracellular ROS was measured using a luminol-based chemiluminescence assay. ROS production was monitored for 30 min at 37°C from neutrophils isolated by the indicated methods and stimulated with classical triggers. Values are represented as delta luminescence. Data are represented as boxplots; whiskers range from the minimum to maximum value and inside each box (25th to 75th percentile) the median is represented; n = 6-9. Individual donors are indicated with distinct symbols. Results were compared using a one-way ANOVA with *post-hoc* Tukey’s multiple comparisons or Kruskal-Wallis test followed by Dunn’s post-test. *P* values are represented as *P< 0.05, **P< 0.01.

To provide a general assessment of the functional consequences associated with the five neutrophil isolation methods, we first investigated the ability of purified neutrophils to generate ROS after stimulation with a range of common triggers, including a protein kinase C activator (PMA), ionophores (A23187, nigericin), bacterial cell wall components (peptidoglycan (PGN), lipopolysaccharide (LPS) or a pro-inflammatory cytokine (TNFα), using a conventional chemiluminescence-based assay (25). Basal ROS production (buffer only) was near to the limit of detection for all the methods evaluated (**Figure 4A**). Upon stimulation with the non-physiological compound PMA and the ionophore A23187, neutrophils from all isolation methods exhibited a robust release of ROS with no significant differences among the isolation methods (**Figures 4B, C**, **Supplementary Figure S2B**). As expected (35), nigericin did not induce any ROS production (**Figure 4D**). The physiological stimuli peptidoglycan induced a robust ROS release from neutrophils of all five methods (**Figure 4E**). However, LPS and TNFα, elicited a robust ROS release only from neutrophils isolated by the S, M, and H/P methods and a marginal release from neutrophils isolated with the methods Pol and D/F (**Figures 4F, G**).

### Neutrophils isolated by methods without RBC lysis undergo lytic cell death after stimulation with biological stimuli

**Figure 5:**
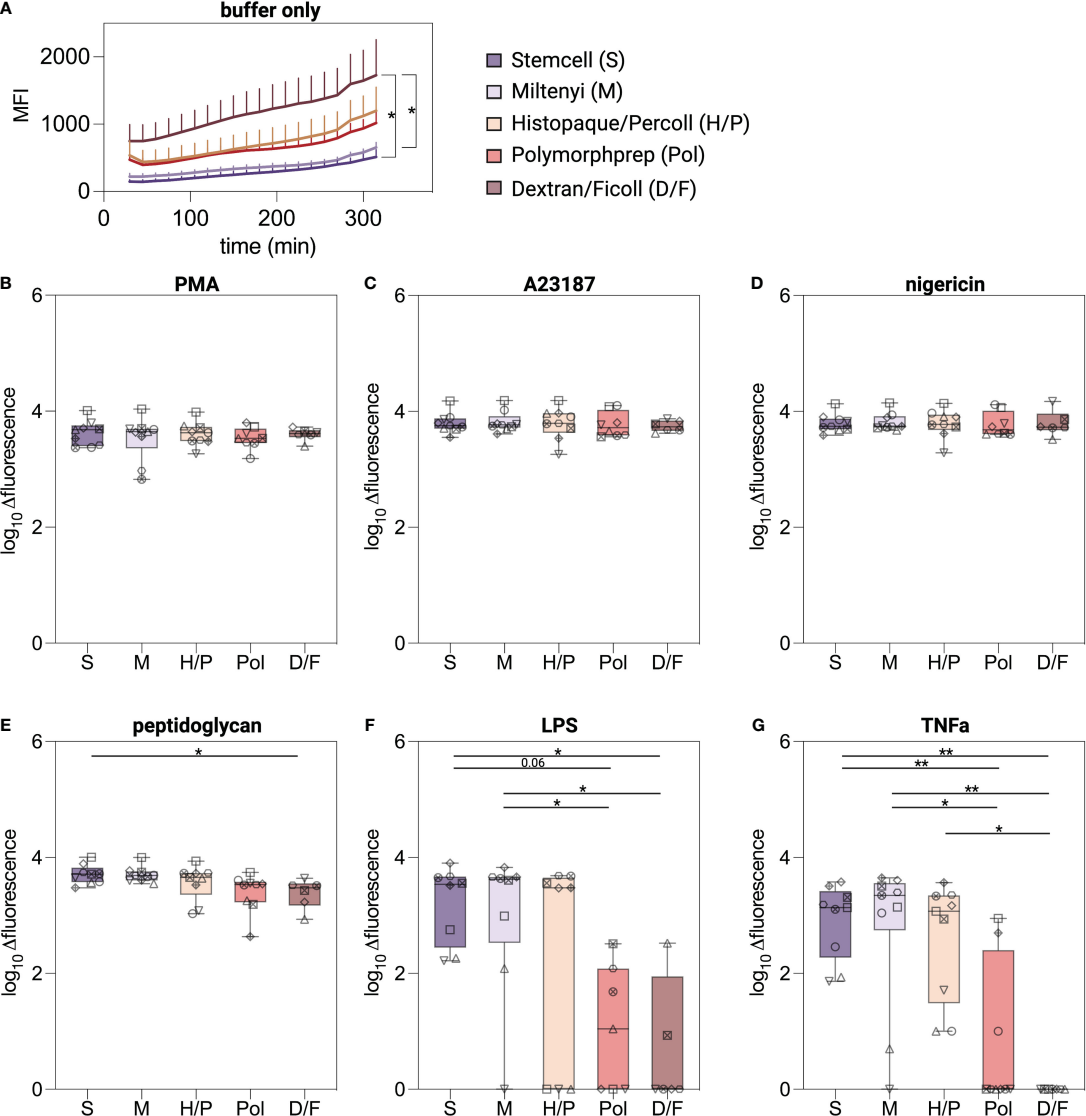
Lytic cell death induced in isolated neutrophils. **(A–G)** Extracellular DNA and DNA of cells with compromised plasma membrane were measured using a plate reader assay. Lytic cell death was monitored for 5 hours at 37°C from neutrophils isolated by indicated methods and stimulated with classical NETosis stimuli. Values are represented as delta fluorescence. Data are represented as boxplots; whiskers range from the minimum to maximum value and inside each box (25th to 75th percentile) the median is represented; n = 6-9. Individual donors are indicated with distinct symbols. Results were compared using a one-way ANOVA with *post-hoc* Tukey’s multiple comparisons or Kruskal-Wallis test followed by Dunn’s post-test. *P* values are represented as *P< 0.05, **P< 0.01.

To evaluate the ability of isolated neutrophils to undergo lytic cell death as an indicator of NETosis, neutrophils were exposed to established NETosis stimuli and cellular breakdown was monitored over a five-hour period using a fluorescence read-out (26). Unstimulated neutrophils isolated with the D/F method displayed higher basal fluorescence values compared to neutrophils isolated with the negative selection kits (**Figure 5A**). PMA, A23187, and nigericin stimulation induced significantly higher levels compared to untreated neutrophils regardless of the isolation method used (**Supplementary Figure S2C**), indicating induction of lytic cell death (**Figures 5B–E**). Peptidoglycan induced similar lytic cell death in S, M and H/P neutrophils, but significantly less in D/F isolated neutrophils (**Figure 5E**). Robust lytic cell death in response to LPS- and TNFα was predominantly observed in neutrophils isolated by the immunomagnetic methods S and M and the density gradient method H/P (**Figures 5F, G**). Pol and D/F density gradient purified neutrophils did not exhibit cell death in response to LPS and TNFα in most donors.

Overall, and in analogy to the ROS production experiments, neutrophils undergo lytic cell death suggestive of NETosis in response to the chemical compound PMA and the calcium ionophore A23187 irrespective of the isolation method used. However, lytic cell death in response to biological stimuli was only observed for neutrophils isolated by low-activating methods.

## Discussion

Studying neutrophils *ex vivo* is challenging due to their short lifespan and reactivity to external triggers. Given the importance of the isolation process in neutrophil research, it is surprising that only few studies have been published comparing different isolation methods with regard to phenotypic and functional consequences (16, 36). Moreover, a comprehensive comparison of several available techniques and kits with unprocessed whole blood neutrophils, is still lacking.

In this study, we performed a comparative analysis of neutrophil purity, yield, viability, activation, and function of the five most commonly employed neutrophil isolation methods in parallel.

Our data show that all isolation procedures yielded comparably viable neutrophils, but with significant differences in purity, recovery, and activation status, as well as time and cost (**Supplementary Table S2**).

Significant disparities emerged when comparing density gradient procedures with immunomagnetic isolation kits. The Pol and D/F density gradient methods yielded purities of ~85% and ~78%, respectively, with lymphocytes and eosinophils as the major contaminants. In contrast, the H/P method achieved ~94% purity, whereas immunomagnetic kits from Stemcell and Miltenyi yielded purities of ~97-98%. These outcomes align with prior studies (37, 38). Securing pure neutrophil isolates is critical for studying gene expression and neutrophil-derived cytokines. Even low levels of contamination can lead to false positives, as demonstrated in previous studies (37, 38). Whereas around 95% purity is suitable for many cases, some studies demand ultrapure neutrophils, requiring alternative protocols (38). Another important difference between immunomagnetic bead separation and density gradient methods with further RBC lysis is the loss of neutrophils during the isolation procedure, which is illustrated by the differences in the absolute yield of neutrophils. Low-density neutrophils (LDNs) are known to be lost during density gradient purifications, which is not the case with the immunomagnetic kits and the H/P method. However, LDNs account for only 4% of circulating neutrophils and can therefore only partially explain the cell loss. It can be anticipated that the ammonium chloride-based RBC lysis and additional washing steps may also contribute to the loss of neutrophils.

Despite the higher performance in purity and quantity of the S, M, and H/P methods, the isolation methods still induced a significant change in neutrophil phenotype when compared to unprocessed whole blood neutrophils, with elevation of surface activation markers CD66b, CD11b, CD63, CD15 and CD64. This activation was more pronounced for methods involving erythrocyte lysis, resulting in CD62L shedding. These phenotypic differences, alongside potential subpopulation depletion, may have implications for downstream functional studies. While no differences were observed with the non-physiological chemical compound PMA or calcium ionophores, physiologically relevant pro-inflammatory mediators (LPS and TNFα) induced more ROS and lytic cell death in immunomagnetically isolated neutrophils and those from the H/P method compared to other density gradient methods. In fact, despite a higher spontaneous NET release compared to immunomagnetic isolation-derived neutrophils, no cell death could be detected after LPS and TNFα stimulation for neutrophils isolated with methods using RBC lysis in most donors. These data are in agreement with a report of Blanter et al, showing that neutrophils isolated with the Stemcell kit responded to LPS and TNFα, but not neutrophils isolated by dextran sedimentation (16).

The potential of LPS and TNFα to induce NETosis remains debated with conflicting results reported in the literature (39–42). Thus, we hypothesize that, among various other factors influencing the outcomes, differences in the activation status of neutrophils at the beginning of an experiment likely contribute to the described inconsistencies regarding certain NETosis stimuli. The exact mechanism by which different density gradient methods result in more activated neutrophils remains unknown, but may involve centrifugation shear stress, duration of the isolation process and thus oxygen exposure (43), or osmotic changes. Indeed, a key difference between protocols that resulted in more quiescent neutrophils was the absence of RBC lysis. Beyond the stress induced by hypertonia, free heme has been shown to induce neutrophil activation (44, 45).

Interestingly, whereas neutrophils isolated with a method including RBC lysis were significantly more activated, they showed a decreased response to “weaker” NETosis stimuli. Continuous stimulation of neutrophils can lead to a phenomenon called “neutrophil exhaustion”, which is characterized by a significant reduction in granule content due to excessive release and an inability to release NETs (46). The diminished response to activating stimuli is attributed to the prior secretion of granules or NETs (47), and may explain why neutrophils isolated through density gradients with subsequent RBC lysis exhibited elevated degranulation markers and failed to respond to more physiological stimuli such as LPS and TNFα.

In conclusion, our study advocates for immunomagnetic beads or the H/P method due to higher purity, yield, and similarity to unprocessed blood neutrophils in terms of phenotype, activation state and functionality. Nevertheless, longer preparation time for density gradient methods and the significantly higher costs (7-fold) associated with immunomagnetic kits warrant consideration (**Supplementary Table S2**). Caution should be taken when studying surface CD32A expression or function, as at least one commercial kit contained Fc blockers that interfered with its detection.

Our results prompt the endorsement of bead-based negative selection kits or density gradient methods devoid of RBC lysis as the new standard for neutrophil isolation from human blood, whereas methods involving erythrocyte lysis should be abandoned. Additional studies are required to evaluate the performance of the different isolation methods using bone marrow as a source, or blood samples from patients with defects in neutrophil maturity and/or granular content, such as leukemia, sepsis or auto-immune diseases.

## Data availability statement

The raw data supporting the conclusions of this article will be made available by the authors, without undue reservation.

## Ethics statement

The studies involving humans were approved by CPP Ile-de-France I” ethics committee. The studies were conducted in accordance with the local legislation and institutional requirements. Human peripheral blood samples were collected from healthy volunteers through the ICAReB platform (Clinical Investigation & Access to Research Bioresources) from the Center for Translational Science, Institut Pasteur. All participants received oral and written information about the research and gave written informed consent in the frame of the healthy volunteers CoSImmGEn cohort (Clinical trials: NCT 03925272).

## Author contributions

VK: Conceptualization, Formal analysis, Investigation, Methodology, Visualization, Writing – original draft. OG: Investigation, Methodology, Writing – review & editing. PB: Funding acquisition, Writing – review & editing. FJ: Conceptualization, Funding acquisition, Methodology, Supervision, Writing – original draft. LC: Conceptualization, Data curation, Methodology, Supervision, Writing – original draft.
