# Supplementary material for: Isolation methods determine human neutrophil responses after stimulation
Source: Front Immunol. 2023 Nov 24;14:1301183. doi: 10.3389/fimmu.2023.1301183 (PMC10704165; doi:10.3389/fimmu.2023.1301183)
Supplement: Supplementary file 1 [file DataSheet_1.docx]

**Supplemental data**

**Supplemental Table1**: Antibodies used for flow-cytometry.

| **Target** | **Antibody-Clone** | **Supplier** |
| --- | --- | --- |
| CD3 | REA613 | Milteny Biotec |
| CD11b | LM2 | BioLegend |
| CD14 | REA599 | Milteny Biotec |
| CD15 | HI95 | BD Pharmingen |
| CD16 | 3G8 | BioLegend |
| CD19 | REA675 | Milteny Biotec |
| CD32A | IV.3 | Stemcell Technologies |
| CD45 | REA196 | Milteny Biotec |
| CD56 | REA747 | Milteny Biotec |
| CD62L | 145/15 | Milteny Biotec |
| CD63 | H5C6 | BioLegend |
| CD64 | 10.1 | BioLegend |
| CD66b | G10F5 | BD Pharmingen |
| CD193 | REA574 | Milteny Biotec |
| CD235 | GA-R2 | BD Bioscience |
| Live/ dead | violet | Thermo Fisher Scientific |

**Supplemental Figure 1**
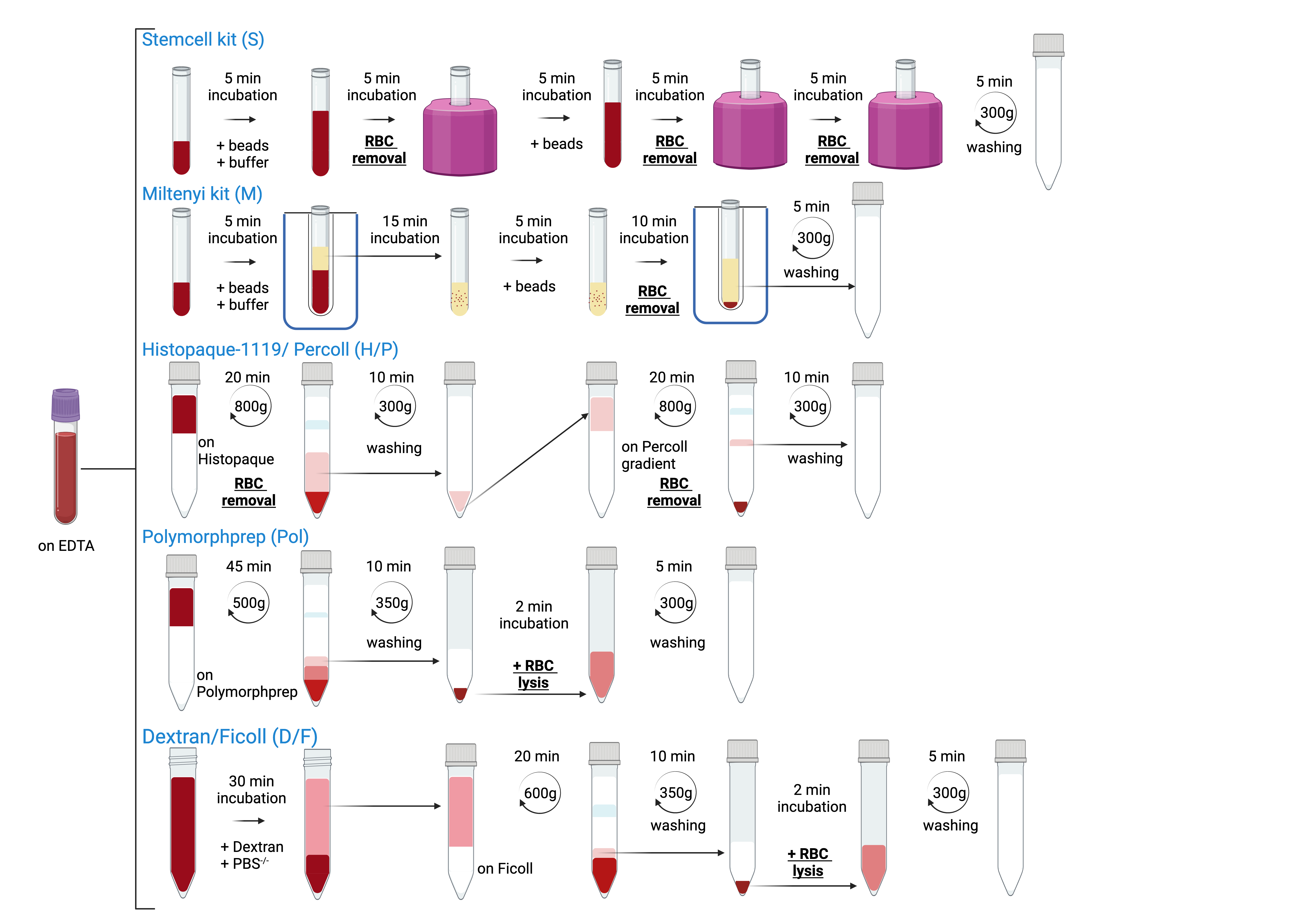


**Supplemental Figure 1.** **Neutrophil isolation methods.** Schematic representation of tested isolation methods. **(A**) Stemcell kit; immunomagnetic isolation, incubation of beads and reagent buffer, followed by incubation in magnetic field. **(B)** Miltenyi kit; immunomagnetic isolation, incubation of beads and reagent buffer, followed by incubation in magnetic field. **(C)** Histopaque/ Percoll; density-gradient method on Histopaque-1119, followed by centrifugation on a Percoll gradient. **(D)** Polymorphprep; density-gradient method on Polymorphprep, followed by RBC lysis. **(E)** Dextran/ Ficoll; incubation of whole blood with dextran, followed by density-gradient method ficoll, followed by RBC lysis. All samples were analyzed by multicolor flow cytometry and plate reader assays. This figure was created with Biorender.com.

**Supplemental Figure 2**


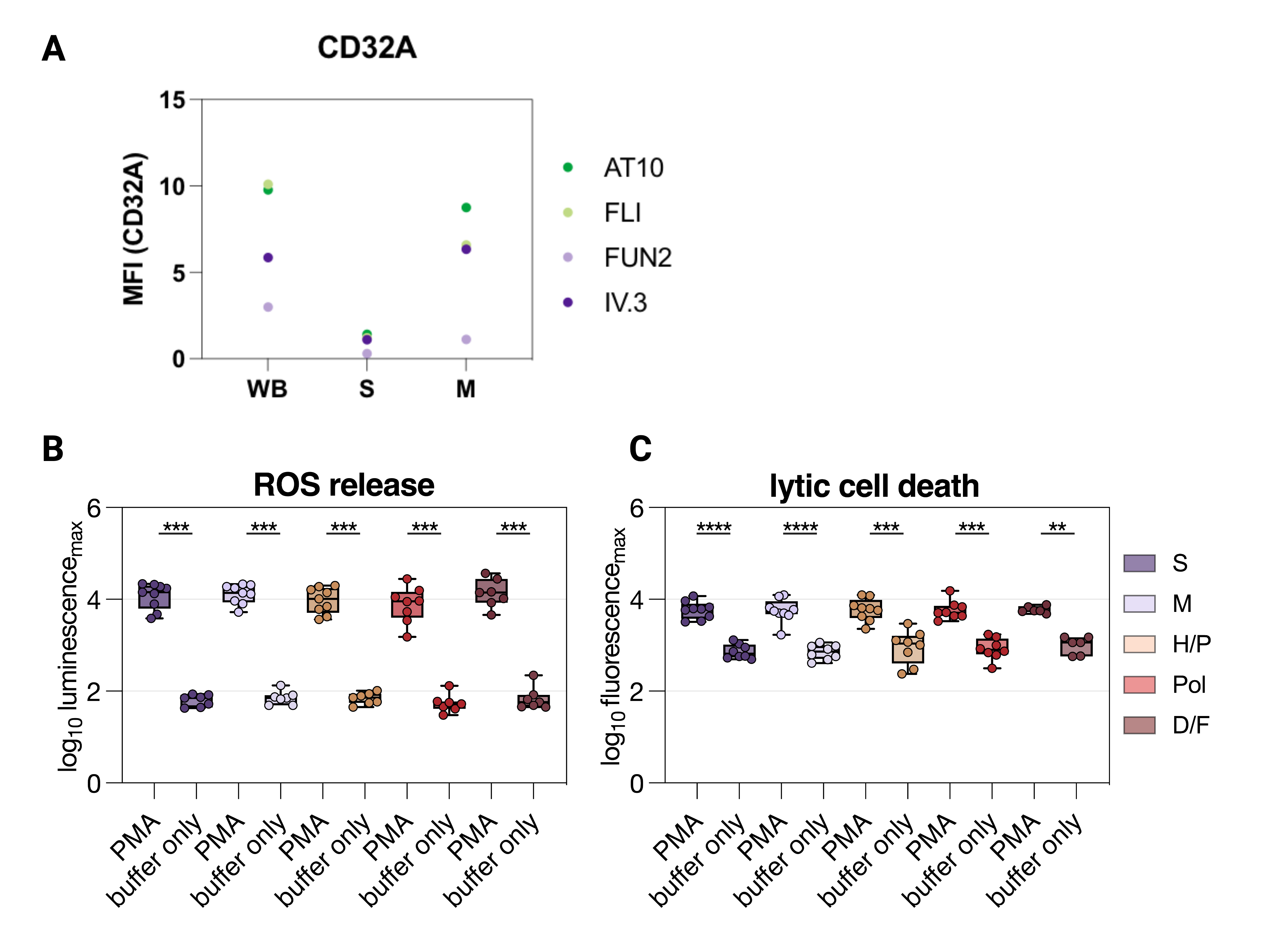


**Supplemental Figure 2**. (**A**) Staining of neutrophils with different anti-CD32A mAbs. **(B)** Representative example that PMA induced a significant ROS production, and (**C**) lytic cell death, compared to unstimulated (buffer only) neutrophils, in all isolation methods used. B/C: Data are represented as boxplots; whiskers range from the minimum to maximum value and inside each box (25th to 75th percentile) the median is represented. Individual donors are indicated with distinct symbols (n = 6-9).

**Supplemental Table 2**. Comparison of results the five different isolation methods.

| **Method** | **Yield (cells/ ml input blood)** | **Purity**  **(%)** | **Fold-change compared to WB neutrophils**  **up down** | | **LPS, TNFα responsiveness**  **ROS lytic**  **death** | | **Time (min)** | **Costs €/ ml WB** |
| --- | --- | --- | --- | --- | --- | --- | --- | --- |
| **S** | 1.71 x 10^6^ ± 0.067 | 97.22 ± 1.77 | CD15 (1.8-fold)  CD64 (3.6-fold) | CD32A (0.3-fold) | ** | ** | 35 | 7,17 |
| **M** | 1.72 x 10^6^ ± 0.076 | 97.91 ± 1.06 | CD11b (1.8-fold)  CD66b (1.8-fold)  CD63 (1.3-fold)  CD15 (1.8-fold)  CD64 (3.5-fold)  CD32A (1.2-fold) |  | ** | ** | 45 | 7,35 |
| **H/P** | 1.15 x 10^6^ ± 0.079 | 94.1 ± 3.61 | CD64 (3.3-fold)  CD32A (1.3-fold) |  | * | * | 70 | 0,88 |
| **Pol** | 0.88 x 10^6^ ± 0.094 | 78.96 ± 6.68 | CD11b (2.2-fold)  CD66b (2.5-fold)  CD63 (2.8-fold)  CD15 (6.8-fold)  CD64 (4.7-fold)  CD32A (1.2-fold) | CD62L (0.5-fold) | - | - | 72 | 0,31 |
| **D/F** | 0.85 x 10^6^ ± 0.084 | 85.12 ± 6.78 | CD11b (2.6-fold)  CD66b (2.8-fold)  CD63 (2.7-fold)  CD15 (5.1-fold)  CD64 (4.7-fold)  CD32A (1.2-fold) | CD62L (0.5-fold) | - | - | 77 | 0,31 |
